# Barriers and facilitators to the implementation of mandatory folate fortification as an evidence-based policy to prevent neural tube defects

**DOI:** 10.1007/s00381-023-05944-x

**Published:** 2023-05-20

**Authors:** Kemel A. Ghotme, Anastasia Arynchyna-Smith, Pedram Maleknia, Vijaya Kancherla, Helena Pachon, Philip J. Van der Wees, Joseph M. Bocchino, Gail L. Rosseau

**Affiliations:** 1 Translational Neuroscience Research Lab, School of Medicine, Universidad de La Sabana, Campus Universitario Puente del Común, Autopista Norte, Km 7, Chia, Colombia; 2 Neurosurgery Department, Fundacion Santa Fe De Bogota, Bogota, DC Colombia; 3 The University of Alabama at Birmingham, Birmingham, AL USA; 4 School of Medicine, University of Alabama, Birmingham, AL USA; 5 Rollins School of Public Health, Emory University, Atlanta, GA USA; 6 The School of Medicine and Health Sciences, The George Washington University, Washington, DC USA; 7 Radboud Institute of Health Sciences, Nijmegen, Netherlands; 8 Department of Neurosurgery, The School of Medicine and Health Sciences, The George Washington University, Washington, DC USA; 9 The Barrow Neurological Institute, Phoenix, AZ USA

**Keywords:** Neural tube defects, Spina bifida, Anencephaly, Mandatory folate fortification, Determinant factors, Implementation, Barriers, Facilitators, Advocacy

## Abstract

**Background:**

Neural tube defects continue to be one of the main congenital malformations affecting the development of the nervous system and a significant cause of disability and disease burden to individuals living with these conditions. Mandatory food fortification with folic acid is, by far, one of the most efficacious, safe, and cost-effective interventions to prevent neural tube defects. However, most countries fail to effectively fortify staple foods with folic acid, impacting public health and healthcare systems and generating dismal disparities.

**Aim:**

This article discusses the main barriers and facilitators for implementing mandatory food fortification as an evidence-based policy to prevent neural tube defects worldwide.

**Methods:**

A comprehensive review of the scientific literature allowed the identification of the determinant factors acting as barriers or facilitators for the reach, adoption, implementation, and scaling up of mandatory food fortification with folic acid as an evidence-based policy.

**Results:**

We identified eight barriers and seven facilitators as determinant factors for food fortification policies. The identified factors were classified as individual, contextual, and external, inspired by the Consolidated Framework for Implementation of Research (CFIR). We discuss mechanisms to overcome obstacles and seize the opportunities to approach this public health intervention safely and effectively.

**Conclusions:**

Several determinant factors acting as barriers or facilitators influence the implementation of mandatory food fortification as an evidence-based policy worldwide. Notoriously, policymakers in many countries may lack knowledge of the benefits of scaling up their policies to prevent folic acid-sensitive neural tube defects, improve the health status of their communities, and promote the protection of many children from these disabling but preventable conditions. Not addressing this problem negatively affects four levels: public health, society, family, and individuals. Science-driven advocacy and partnerships with essential stakeholders can help overcome the barriers and leverage the facilitators for safe and effective food fortification.

## Introduction

Thousands of children can be saved from being born with a neural tube defect (NTD). NTDs are a set of severe congenital malformations of the central nervous system due to an absent, incomplete, or impaired closure of the neural tube in the embryonic stage, leading to significant neurological deficits, disability, and related complications in patients affected with these conditions [1]. NTDs constitute a substantial cause of pregnancy termination, stillbirths, mortality, morbidity, and long-term disability.

Annually, up to three of every 1000 children can be born with an NTD, including anencephaly or spina bifida, with a global estimated rate of 300,000 new cases per year worldwide [2, 3]. In more than 70% of the cases, the cause is a maternal folic acid deficiency or insufficiency [4]. Although other risk factors have been identified, including gestational diabetes, genetic abnormalities, and teratogenic exposure to medications and other physical or chemical agents [5], folic acid-sensitive NTDs continue to be, by far, one of the main preventable congenital malformations. Despite substantial efforts to understand the genetics, pathophysiology, and surgical treatment of NTDs, the natural history of these conditions continues to exhibit high morbidity and marked impairment of the quality of life of affected patients [6].

Except for children with lethal malformations, patients with NTDs can undergo successful corrective surgery after birth, or during the intrauterine stage, in countries with installed healthcare capacity to perform such procedures. Notwithstanding, and despite successful neurosurgical treatments, these patients might face different long-term health issues in physical, cognitive, psychological, and social areas, requiring additional surgeries and several treatments and aids during their lifetime. Furthermore, the calculated direct and indirect care costs for individuals with NTDs are enormous, which ensures profound inequities and disparities in disease burden, especially for low- and middle-income countries [6].

With this landscape, the ideal scenario is the primary prevention of NTDs. Mandatory food fortification (MFF) is a successful public health evidence-based policy to prevent folic acid-sensitive NTDs, which has been available for several decades [7]. As a public health intervention, scientific evidence has shown that food fortification is a practical, safe, feasible, and cost-effective policy. It also decreases costs associated with healthcare and helps countries achieve their sustainable development goals [8, 9, 11]. Despite these facts, only 92 countries worldwide have adopted a mandatory fortification policy for cereal grains. From those, only 63 countries include folic acid in their fortification standard, and the majority focus solely on one staple food, wheat flour [4, 10–12].

MFF policies prevented nearly 62,000 cases of all preventable NTDs as of 2020, accounting for only 22% of the NTDs that could be prevented. There are an additional 200,000 preventable cases in more than 100 countries that do not implement MFF with folic acid [10–12].

The literature points to different determinant factors acting as barriers or facilitators for the reach, adoption, implementation, and scaling up of MFF with micronutrients, including folic acid, as an evidence-based policy. They comprise individual and contextual factors, internal or external, that may vary according to specific circumstances. However, there is scarce information in the literature regarding theories, models, frameworks, or knowledge translation interventions that address how to approach those factors productively.

This article discusses the main determinant factors for implementing mandatory food fortification policies and the role of neurosurgical advocacy in overcoming those barriers and leveraging the facilitators to effectively and successfully implement MFF to prevent NTDs.

## Barriers

**Fig. 1:**
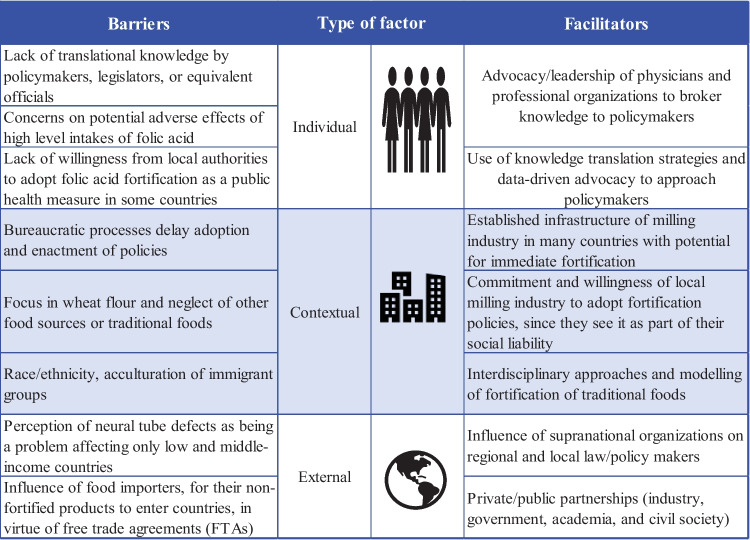
Critical determinant factors acting as barriers and facilitators to adopting mandatory food fortification policies. Note: factor classification inspired by the Consolidated Framework for Implementation of Research (CFIR)

Figure 1 summarizes the main barriers to the implementation of MFF policies. Despite substantial evidence of the safety, efficacy, and effectiveness of MFF as a successful public health intervention, one of the evident barriers in the literature is a lack of willingness from local authorities in many countries to either enact regulations for this evidence-based policy or to provide sufficient oversight to ensure that industry and importers follow the rules for mandatory folic acid fortification [6, 13]. One potential reason is the concern that exposure to high doses of folic acid might cause an increased risk of different disorders.

Higher-than-normal serum folate levels have been associated with health issues such as cancer, asthma, cognitive problems, twin pregnancy, and autism [14]. Excessive folic acid intake may mask a vitamin B12 deficiency, potentially resulting in neurologic damage [13]. However, more recent scientific evidence does not confirm these risks [15, 16]. Moreover, the evidence points in the opposite direction since no singular study has enough level of evidence to recommend against food fortification on a massive scale.

Although masking of vitamin B12 deficiency in older adults with macrocytic anemia has been described if they are only treated with folate and not folic acid [13], in modern practice, it is unlikely that vitamin B12 deficiency masking would happen [15]. The available evidence indicates that folic acid intakes of up to 1 mg/day, the adult upper level of intake, will not mask the diagnosis of vitamin B12 deficiency [15]. At the same time, the upper level’s relevance for younger age groups, particularly children, is unclear because vitamin B12 deficiency is rare in the pediatric population [13]. Moreover, Wald et al. and Pachon et al. have stated that there is no scientific basis for setting an upper level of intake for folate and proposed that the upper level should be eliminated since it acts as a barrier to large-scale fortification (Pachón et al. 2021; [16].

A rare condition causes slow processing of folates [7], however, it does not lead to toxic serum folate levels in individuals receiving folic acid supplements at the recommended doses since folic acid is a water-soluble vitamin excreted in the urine when it reaches excessive serum concentration. Although there is the potential for increased folic acid intake to interfere with certain medications, the available scientific evidence does not demonstrate any clinically significant interaction with therapeutic medicines from folate intakes up to 1 mg/day (Choi et al. 2006).

The ambiguous role of synthetic folic acid in promoting subclinical cancers, mainly colorectal cancer, has led to the hesitation of some countries, mainly in Europe, to introduce a public health intervention for MFF [17]. Nevertheless, the increased incidence of colorectal cancer is more attributable to improved screening for that type of neoplasm. Quite the opposite, there is evidence of a protective effect for pancreatic cancer with increasing dietary folate intake [18].

One study discusses folic acid fortification’s role in a higher risk of malaria in African countries [19]. However, this finding has been controversial since folic acid supplements usually contain iron, whose high levels reportedly increase the risk of malaria,meanwhile, other studies have shown a protective effect of folates against that parasitic illness. The authors propose a dose-dependent effect, whereby intake of low doses of folic acid (which corresponds to the daily intake from food fortification) would have a marginal impact on malaria.

Another source of resistance to MFF policies is the belief that they limit consumers’ choice regarding opting for non-fortified products; however, this is not the main issue in many low- and middle-income countries, where poverty remains the limiting factor to access processed foods for most of the population (Allen et al. 2006).

Other aspects, including perceived costs of fortification by the food industry, the concept that NTDs are a problem that only pertains to low and middle-income countries, and socio-political reasons, might also play a role. Only three countries in Europe (Moldova and Kosovo, and more recently, the UK) have embraced mandatory folic acid fortification (DEFRA 2021; [4] Global Fortification Data Exchange 2022), resulting in more than a thousand pregnancies affected by preventable spina bifida and anencephaly every year in that continent [10–12]. The reasons for the endurance of this prevalence are multifactorial. For instance, a study in Italy addressed the transition in dietary habits affecting the Mediterranean diet, previously shown to be protective against NTDs, to a more “North American” diet as one potential cause for this phenomenon [14]. On the other hand, increasing migration of people from African countries and Eastern Europe could have also changed the landscape of dietary habits and access to quality food in some communities living in Southern Europe. Fisher et al. argue that folic acid supplements alone are insufficient to decrease the incidence of NTDs,therefore, efforts for food fortification, among others, are needed [14]. In 2021, the UK mandated adding folic acid to wheat flour, previously fortified with other micronutrients. However, the amount of folic acid is still being stipulated (DEFRA 2021).

In a global context, despite countries having existing policies for fortification of cereal grains with folic acid, most of them focus only on one staple (mainly wheat flour) [4], leaving groups of people who favor other food sources (such as maize flour or rice) in their diet without the benefit of getting folic acid-enriched products. In some regions, vulnerable communities do not access industrially processed, fortified wheat flour and derivate products [20], Marchetta et al. 2015; [21] and base their diet on rice, corn masa, yucca (cassava), teff, or quinoa for geographic, historical, cultural, or ethnic reasons or because those grains constitute the only staple. Therefore, those countries with existing policies can benefit from updating and scaling up MFF policies to include other staples with the recommended guidelines for folic acid while optimizing surveillance and ensuring the sustainability of existing policies.

Different dietary habits rooted in local or national cultures may also act as barriers leading to a low reach of MFF as an impactful measure to prevent NTDs. For instance, Mexican American women, a vulnerable immigrant population in the USA whose offspring have a high incidence of NTDs, rely on non-fortified products such as corn masa as the main component of their traditional recipes [20]. This cultural practice, along with other potential factors such as race/ethnicity and acculturation, is associated with lower folate intake and low serum folate levels among women of reproductive age in that group [20, 22]. Hence, the fortification of wheat flour has a weak influence on these communities since the basis of their dietary habits does not include wheat flour products but other non-fortified cereal grains such as non-fortified maize flour, a common ingredient in corn masa and other traditional recipes made with locally grown or imported grains. In that sense, studies modeling fortification of traditional foods like corn masa found a positive potential to selectively increase total folic acid intake among Mexican American women without exceeding the tolerable upper intake level for folic acid [21, 23].

Finally, most countries with MFF policies in place have systems for surveilling implementation. However, documentation of compliance with those policies, the roles and responsibilities between agencies, the cost of regulating fortification, and enforcement strategies, are often lacking [24].

## Facilitators

The literature also suggests facilitators for implementing MFF policies, summarized in Fig. 1.

The first facilitator is the compelling evidence supporting that regulations that enforce mandatory folic acid fortification of one or more grain cereals and their derivates induce a significant decrease in the incidence of NTDs and their associated morbidity and mortality (Atta et al. 2016; Garrett and Bailey 2018; Kancherla et al. 2014).

As an evidence-based policy, MFF is practical since it does not require women to change behaviors (such as taking supplements) to improve their periconceptional folate status (Martinez et al. 2018, 2021; Pachón et al. 2013). It is also safe, given that programs implemented in many countries have no adverse consequences (Field et al. 2018). MFF is feasible since over a hundred countries already have mandatory fortification with micronutrients of different foods, including maize flour (19 countries), oil (34 countries), rice: (8 countries), salt (126 countries), and wheat flour (91 countries) (Global Fortification Data Exchange 2022). Besides, it is feasible because countries with existing industrial milling infrastructure can immediately fortify staple foods and prevent more than 50,000 cases annually without costly investments [25]. Last, and most importantly, it is cost-effective because fortifying food is inexpensive and saves lives and millions in resources and efforts (CDC 2020b).

Supranational policies encourage local governments to achieve Sustainable Development Goals by 2030, including ending hunger and improving the population’s health status, as is the case for preventing NTDs [26]. On the other hand, private/public partnerships, adequate monitoring, and quality control are among the main components of successful staple food fortification programs. They might also be critical elements for the sustainability of those programs [27].

One seminal paper highlights the central role that neurosurgeons and organized neurosurgery can play in advocating for a more comprehensive, global-scale folate fortification to avoid the most common and severe birth congenital malformation that affects the human nervous system due to their accumulated experience dealing with these conditions and their high standing in society [6]. These authors propose that assertive, proactive, informed advocacy for folate fortification should be integral to the neurosurgical approach to NTDs. Furthermore, they recommend eight steps to materialize this advocacy, as listed below:

1. Neurosurgeons and neurosurgical professional organizations must serve as powerful advocates for MFF with folic acid.
2. Forming partnerships with local and international colleagues to advance basic and clinical research.
3. Supporting improved registry and surveillance efforts on a local and global scale.
4. Advocating for increased prenatal screening of NTDs.
5. Supporting the establishment of comprehensive countrywide centers of excellence to integrally approach NTDs through a combination of advocacy, international collaboration, and funding.
6. Working to establish and expand partnerships between their institutions and existing NTD centers in developing countries.
7. NTD advocacy organizations and organized neurosurgical groups must expand the availability of multidisciplinary conferences on NTD prevention and multidisciplinary management across the world.
8. International initiatives can provide country-level information on NTD prevalence and local prevention and can serve as partners to effect significant change.

As key opinion leaders, neurosurgeons can act as knowledge brokers to facilitate the adoption, implementation, and scaling up of MFF as an evidence-based policy to prevent NTDs. The International Society for Pediatric Neurosurgery (ISPN) constituted a Spina Bifida Global Taskforce with the multi-national and multidisciplinary collaboration of individuals and organizations interested in the primary prevention of major folic acid-sensitive NTDs. This organization stated that pediatric neurosurgeons are essential data-driven advocates for MFF policies, with the potential to spearhead the protection of thousands of children in all countries [28].

## Overcoming barriers and leveraging facilitators: the role of neurosurgical advocacy

Evidence-based policies are public health actions informed by a consideration of the scientific evidence, but the decisions made will depend on determinant factors and prevailing values and priorities; therefore, this process often requires the interplay of advocacy, lobbying, and more complex social and political negotiations than only appraising evidence and formulating recommendations (Rychetnik et al. 2004). In this set, organized neurosurgery plays an essential role in advocating for impactful evidence-based policies.

In a scientific meeting held in Bolivia in 2006, the Latin American Association for Pediatric Neurosurgery (ASOLANPED) promulgated the Declaration of Santa Cruz. This document reinforced the high incidence of NTDs in Latin American countries as a social problem preventable by implementing MFF. The declaration also recommended that the region’s governments support this policy and consider approaching other factors related to NTDs in the specific Latin American context [29].

The liaison committee between the WHO and the World Federation of Neurosurgical Societies (WFNS) works to advance access to quality care for neurosurgical patients globally [30]. National and regional neurosurgical societies play an important role in advancing the global neurosurgical agenda, including ways to influence public policies impacting the incidence and effects of conditions that affect the human nervous system. In low- and middle-income countries, contributions include advocating for compiling information regarding the neurosurgical disease burden and accurate reporting of human health resources and may also include evaluation of resource-stratified interventions, policies, and equipment [30].

In 2021, the International Society for Pediatric Neurosurgery (ISPN) recommended that all governments enact policies for MFF with folic acid of centrally produced staples to provide almost all women of reproductive age who eat fortified foods with at least an additional 150 μg/day of folic acid, according to the WHO recommended guidelines [28].

Partnerships joining efforts from multiple stakeholders are crucial since they combine diverse expertise and perspectives. The Global Alliance for the Prevention of Spina Bifida F (GAPSBiF), a multidisciplinary coalition of neurosurgeons, pediatricians, geneticists, epidemiologists, food scientists, and fortification policy experts, was formed to advocate for MFF of staple foods worldwide [31].

A recent call to action from the scientific community led by GAPSBiF, published in The Lancet Global Health, urges the World Health Assembly to pass a resolution for universal mandatory folic acid fortification [10–12]. Such a resolution can accelerate the slow pace of NTD prevention globally and assist countries in reaching their 2030 Sustainable Development Goals on decreasing child mortality and promoting health equity.

## Conclusions

Neurosurgical conditions, specifically congenital malformations of the central nervous system, are usually devastating, and their care is highly demanding in terms of costs and effort. However, the involvement of neurosurgeons in public health initiatives is scarce. Their participation, advocacy, and lobbying can be impactful in promoting evidence-based policies and integrating the neurosurgical burden into national health planning systems [32].

NTDs can diminish the affected individuals’ survival, health, and quality of life and impact families and society. MFF, the most effective measure to prevent NTDs, has been available for more than three decades, but still, more than one hundred countries fail to fortify food with folic acid as an evidence-based policy. The cost of inaction is profound and disproportionately impacts susceptible populations worldwide, with a more significant impact in low-income and middle-income countries.

Separate disciplines, including medicine, nutrition, pediatrics, public health, and epidemiology, have identified gaps and opportunities for implementing strategies to adopt MFF as a public health intervention. However, there is a lack of cross-disciplinary research to move forward and include relevant stakeholders in the dialog, aiming to scale up this evidence-based policy and reach thousands of communities that may benefit from the intervention.

Important determinant factors acting as barriers or facilitators for implementing MFF as an evidence-based policy are evident in the literature. Furthermore, the literature allows inferring that policymakers in many countries may lack knowledge of the benefits of scaling up their policies for MFF to prevent folic acid-sensitive NTDs, improve the health status of their communities, and promote the protection of a large number of children from these disabling but preventable conditions. Not addressing this problem negatively affects four levels: public health, society, family, and individuals.

Future exploration of this problem might consider theories, models, and frameworks for materializing the role of neurosurgeons and neurosurgical societies and generating knowledge translation strategies to leverage science-driven advocacy. The ultimate goal is to ensure MFF’s reach, adoption, implementation, scaling up, and sustainability as a robust evidence-based policy to prevent NTDs and promote a healthy start to many children’s life.

## Data Availability

The full dissertation is available as gray literature at https://hsrc.himmelfarb.gwu.edu/smhs_crl_dissertations/18/.

## Abbreviations

ASOLANPED: Latin American Association for Pediatric Neurosurgery
GAPSBiF: Global Alliance for the Prevention of Spina Bifida F
ISPN: International Society for Pediatric Neurosurgery
MFF: Mandatory food fortification
NTDs: Neural tube defects

## References

[1] Greene ND, Copp AJ (2014) Neural tube defects. Annual review of neuroscience. Annualreviews.Org. https://www.annualreviews.org/doi/abs/10.1146/annurev-neuro-062012-170354 10.1146/annurev-neuro-062012-170354 PMC4486472 25032496

[2] Blencowe H, Kancherla V, Moorthie S, Darlison MW, Modell B (2018). Estimates of global and regional prevalence of neural tube defects for 2015: a systematic analysis. Ann N Y Acad Sci.

[3] Zaganjor I, Sekkarie A, Tsang BL, Williams J, Razzaghi H, Mulinare J, Sniezek JE, Cannon MJ, Rosenthal J (2016). Describing the prevalence of neural tube defects worldwide: a systematic literature review. PLoS ONE.

[4] FFI Food Fortification Initiative (2022) Global progress of industrially milled cereal grain fortification- food fortification initiative. http://www.ffinetwork.org/global_progress/

[5] Mitchell LE (2005) Epidemiology of neural tube defects. Am J Med Genet Semin Med Genet 135 C(1):88–94. 10.1002/ajmg.c.30057 10.1002/ajmg.c.30057 15800877

[6] Estevez-Ordonez D, Davis MC, Hopson B, Arynchyna A, Rocque BG, Fieggen G, Rosseau G, Oakley G, Blount JP (2018) Reducing inequities in preventable neural tube defects: the critical and underutilized role of neurosurgical advocacy for folate fortification. Thejns.Org. https://thejns.org/focus/view/journals/neurosurg-focus/45/4/article-pE20.xml 10.3171/2018.7.FOCUS18231 PMC8500574 30269587

[7] Crider KS, Bailey LB, Berry RJ (2011). Folic acid food fortification-its history, effect, concerns, and future directions. Nutrients.

[8] Hoddinott J (2018). The investment case for folic acid fortification in developing countries. Ann N Y Acad Sci.

[9] Högler W, Aguiar M, Kiely M, Tulchinsky T (2016). Consensus recommendations for prevention of nutritional rickets: food fortification and micronutrient supplements for global health. AIMS Public Health.

[10] Kancherla V, Botto LD, Rowe LA, Shlobin NA, Caceres A, Arynchyna-Smith A, Zimmerman K, Blount J, Kibruyisfaw Z, Ghotme KA, Karmarkar S, Fieggen G, Roozen S, Oakley GP, Rosseau G, Berry RJ (2022). Preventing birth defects, saving lives, and promoting health equity: an urgent call to action for universal mandatory food fortification with folic acid. Lancet Glob Health.

[11] Kancherla V, Roos N, Walani SR (2022) Relationship between achieving Sustainable Development Goals and promoting optimal care and prevention of birth defects globally. In Birth Defects Res (Vol. 114, Issue 14, pp. 773–784). John Wiley and Sons Inc. 10.1002/bdr2.2055 10.1002/bdr2.2055 35776686

[12] Kancherla V, Wagh K, Priyadarshini P, Pachón H, Oakley GP (2022). A global update on the status of prevention of folic acid-preventable spina bifida and anencephaly in year 2020: 30-year anniversary of gaining knowledge about folic acid's prevention potential for neural tube defects. Birth Defects Res.

[13] Mills JL (2017). Strategies for preventing folate-related neural tube defects supplements, fortified foods, or both?. In JAMA - Journal of the American Medical Association.

[14] Fischer M, Stronati M, Lanari M (2017) Mediterranean diet, folic acid, and neural tube defects. Ital J Pediatr 43(1). 10.1186/s13052-017-0391-7 10.1186/s13052-017-0391-7 PMC5561554 28818086

[15] Berry RJ (2019) Lack of historical evidence to support folic acid exacerbation of the neuropathy caused by vitamin B12 deficiency. In Am J Clin Nutrition 110(3):554–561. Am J Clin Nutr. 10.1093/ajcn/nqz089 10.1093/ajcn/nqz089 PMC6785032 31187858

[16] Wald NJ, Morris JK, Blakemore C (2018) Public health failure in the prevention of neural tube defects: time to abandon the tolerable upper intake level of folate. In Public Health Rev (Vol. 39). EHESP Presses. 10.1186/s40985-018-0079-6 10.1186/s40985-018-0079-6 PMC5809909 29450103

[17] Smith AD, Kim YI, Refsum H (2008) Is folic acid good for everyone? Academic Oup Com. https://academic.oup.com/ajcn/article-abstract/87/3/517/4633283 10.1093/ajcn/87.3.517 18326588

[18] Jägerstad M (2012). Folic acid fortification prevents neural tube defects and may also reduce cancer risks. In Acta Paediatrica, Int J Paediatr.

[19] Nzila A, Okombo J, Hyde J (2016). Malaria in the era of food fortification with folic acid. Food Nutr Bull.

[20] Hamner HC, Cogswell ME, Johnson MA (2011). Acculturation factors are associated with folate intakes among Mexican American women. J Nutr.

[21] Tinker SC, Cogswell ME, Hamner HC, Berry RJ (2012) Usual folic acid intakes: a modelling exercise assessing changes in the amount of folic acid in foods and supplements, National Health and Nutrition Examination Survey, 2003–2008. Public Health Nutrit 10.1017/S1368980012000638 22455758

[22] Marchetta CM, Hamner HC (2016). Blood folate concentrations among women of childbearing age by race/ethnicity and acculturation, NHANES 2001–2010. Matern Child Nutr.

[23] Hamner HC, Tinker SC, Flores AL, Mulinare J, Weakland AP, Dowling NF (2013) Modelling fortification of corn masa flour with folic acid and the potential impact on Mexican-American women with lower acculturation. Public Health Nutr 10.1017/S1368980012004582 PMC10271608 23113948

[24] Marks KJ, Luthringer CL, Ruth LJ, Rowe LA, Khan NA, De-Regil LM, López X, Pachon H (2018). Review of grain fortification legislation, standards, and monitoring documents. Glob Health Sci Pract.

[25] Kancherla V (2018). Countries with an immediate potential for primary prevention of spina bifida and anencephaly: mandatory fortification of wheat flour with folic acid. Birth Defects Res.

[26] Kancherla V, Redpath B, Oakley GP (2019) Reductions in child mortality by preventing spina bifida and anencephaly: implications in achieving Target 3.2 of the Sustainable Development Goals in developing countries. Birth Defects Res. 10.1002/bdr2.1362 10.1002/bdr2.1362 30070775

[27] Martorell R, de Romaña DL (2017). Components of successful staple food fortification programs: lessons from Latin America. In Food Nutri Bull.

[28] Caceres A, Blount JP, Messing-Jünger M, Chatterjee S, Fieggen G, Salomao JF (2021). The International Society for Pediatric Neurosurgery resolution on mandatory folic acid fortification of staple foods for prevention of spina bifida and anencephaly and associated disability and child mortality. Childs Nerv Syst.

[29] Dabdoub CF, Dabdoub CB, Villavicencio R, Quevedo G (2014). Como lo hago yo: mielomeningocele en bolivia. Surg Neurol Int.

[30] Rosseau G, Johnson W, Park K, Arráez Sánchez M, Servadei F, Vaughan K (2018) Global neurosurgery: continued momentum at the 72nd World Health Assembly. Neurosurg Focus 45(4):E18. https://thejns.org/view/journals/j-neurosurg/132/4/article-p1256.xml 10.3171/2018.7.FOCUS18295 30269578

[31] Shlobin NA, Roach JT, Kancherla V, Caceres A, Ocal E, Ghotme KA, Lam S, Park KB, Rosseau G, Blount JP, Boop FA, GAPSBiF (the Global Alliance for the Prevention of Spina Bifida-F) (2022) The role of neurosurgeons in global public health: the case of folic acid fortification of staple foods to prevent spina bifida. J Neurosurg: Pediatr 1–8. 10.3171/2022.9.PEDS22188 10.3171/2022.9.PEDS22188 36334286

[32] Veerappan VR, Gabriel PJ, Shlobin NA, Marks K, Ooi SZY, Aukrust CG, Ham E, Abdi H, Negida A, Park KB, El Ouahabi A (2022). Global neurosurgery in the context of global public health practice-a literature review of case studies. World Neurosurg.

[33] Allen L, De Benoist B, Dary O, Hurrell R (2006) Guidelines on food fortification with micronutrients A T. Inunscn.org. http://www.unscn.org/layout/modules/resources/files/fortification_eng.pdf

[34] DEFRA (2021) Amending the Bread and Flour Regulations 1998 and the Bread and Flour Regulations (NorthernIreland) 1998 - Defra - Citizen Space. https://consult.defra.gov.uk/food-compositional-standards/bread-and-flourconsultation-2022/

[35] Global Fortification Data Exchange (2022) Global Fortification Data Exchange | GFDx. https://fortificationdata.org/interactive-map-fortification-legislation/

[36] Atta CAM, Fiest KM, Frolkis AD, Jette N, Pringsheim T, St Germaine-Smith C, Rajapakse T, Kaplan GG, Metcalfe A (2016) Global birth prevalence of spina bifida by folic acid fortification status: A systematic review and meta-analysis. Am J Public Health 106(1):e24–e34. American Public Health Association Inc. 10.2105/AJPH.2015.302902 10.2105/AJPH.2015.302902 PMC4695937 26562127

[37] Garrett GS, Bailey LB (2018) A public health approach for preventing neural tube defects: folic acid fortification and beyond. Ann NY Acad Sci 1414(1):47–58. 10.1111/nyas.13579 10.1111/nyas.13579 29450891

[38] Kancherla V, Druschel CM, Oakley GP (2014) Population-based study to determine mortality in spina bifida: New York State congenital malformations registry, 1983 to 2006. Birth Defects Research Part A - Clinical and Molecular Teratology. 10.1002/bdra.23259 10.1002/bdra.23259 24975407

[39] Martinez H, Weakland AP, Bailey LB, Botto LD, De-Regil LM, Brown KH (2018) Improving maternal folate status to prevent infant neural tube defects: working group conclusions and a framework for action. Ann NY Acad Sci 1414(1):5–19. 10.1111/nyas.13593 10.1111/nyas.13593 29532514

[40] Martinez H, Pachón H, Kancherla V, Oakley GP (2021) Food Fortification With Folic Acid for Prevention of Spina Bifida and Anencephaly: The Need for a Paradigm Shift in Evidence Evaluation for Policy-Making. Am J Epidemiol 190(10):1972–1976. 10.1093/aje/kwab061 10.1093/aje/kwab061 PMC8485149 33728445

[41] Marchetta CM, Devine OJ, Crider KS, Tsang BL, Cordero AM, Qi YP, Guo J, Berry RJ, Rosenthal J, Mulinare J, Mersereau, P, Hamner, HC (2015) Assessing the association between natural food folate intake and blood folate concentrations: A systematic review and Bayesian meta-analysis of trials and observational studies. Nutrients. 10.3390/nu7042663 10.3390/nu7042663 PMC4425166 25867949

[42] Pachón H, Kancherla V, Handforth B, Tyler V, Bauwens L (2013) Folic acid fortification of wheat flour: A cost-effective public health intervention to prevent birth defects in Europe. Nutr Bull 38(2):201–209). 10.1111/nbu.12023

[43] Pachón H, Reynolds B, Duong M, Tsang BL, Childs L, Luthringer CL, Kang Y, Vasta FC, Codling K (2021) The potential contribution of fortified maize flour, oil, rice, salt, and wheat flour to estimated average requirements and tolerable upper intake levels for 15 nutrients in 153 countries. Nutrients 13(2):1–14. 10.3390/nu13020579 10.3390/nu13020579 PMC7916358 33572488

[44] Field M, Sciences, P. S.-A. of the N. Y. A. of, 2018, U (2018) Safety of folic acid. Ncbi.Nlm.Nih.Gov. https://www.ncbi.nlm.nih.gov/pmc/articles/PMC5849489

[45] CDC (2020b) Key Findings: Cost savings of spina bifida prevention after folic acid fortification in the United States | CDC. https://www.cdc.gov/ncbddd/folicacid/features/keyfinding-folicacid-impact.html

[46] Rychetnik L, Hawe P, Waters E, Barratt A, Frommer M (2004) A glossary for evidence based public health. J Epidemiol Community Health 58(7):538–545. BMJ Publishing Group Ltd. 10.1136/jech.2003.011585 10.1136/jech.2003.011585 PMC1732833 15194712

[47] Choi J, Yates Z, Veysey M, … Y H-P nutrition and 2014 U (2006) Contemporary issues surrounding folic acid fortification initiatives. Ncbi.Nlm.Nih.Gov.https://www.ncbi.nlm.nih.gov/pmc/articles/PMC4287316/10.3746/pnf.2014.19.4.247 PMC4287316 25580388

[48] Ghotme KA (2023) The NeuroAdvocacy Toolkit: A Knowledge Translation Strategy to Strengthen Food Fortification Policies to Prevent Neural Tube Defects in Latin American Countries. A Mixed-Method Study.

